# A method of building information modelling implementation for structural engineering firms

**DOI:** 10.1016/j.mex.2024.102685

**Published:** 2024-04-01

**Authors:** Ibrahim F. Varouqa, Moawiah A. Alnsour

**Affiliations:** Department of Civil Engineering, Isra University, Faculty of Engineering, Amman, Jordan

**Keywords:** Resources, Firms, BIM, SEF, Buffalo, Vulture, BIM adoption, Planning, *Building Information Modelling Method*

## Abstract

Building Information Modelling (BIM), a new concept and methodology, has received much attention lately. Various Structural Engineering Firms (SEFs) have observed substantial competitive benefits after its deployment. BIM offers a wide range of advantages, but its capacity has not even been completely exploited. The challenges of firm implementation, a procedure that necessitates significant alterations in firm business structure, are a major factor in this. However, there hasn't been much in-depth research on the assessment and integration of the research around the application of BIM in firms. To address the planning phase's complexity, which makes implementing BIM in these workplaces challenging, this article provides a framework for BIM deployment in the SEF. Additionally, a brand-new hybrid African Buffalo and African Vulture Optimization (AB-AVO) has been created to assess the state of technology. The technique suggested for BIM execution within SEF obviously and effectively recognises the firm's expectations and resources, sets out the needs required to create the BIM technique, and offers technical and clinical suggestions for monitoring and planning the execution. It is categorised by resource utilization, versatility, and adaptability.

• This paper introduces a new concept and methodology of using BIM
• The technique suggested for BIM execution within SEF obviously and effectively recognises the firm's expectations and resources
• The method establishes guidelines for structural firm to adopt BIM in their monitoring, and planning the execution

## Graphical Abstract

**Figure:**
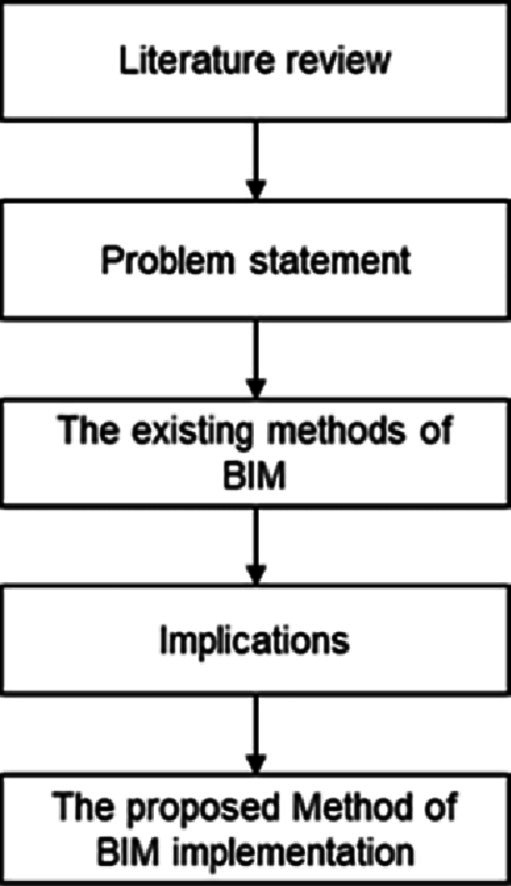

Specifications Table

**Table:** 

Subject area:	*Building Information Modelling*
Specific subject:	*Construction Management*
Method name:	*Building Information Modelling Method*
Name and reference of original method:	*Not applicable*
Recourse Availability:	*Not applicable*

## Background

The structural layout of a construction or infrastructure project has been a complicated and dynamic process, constantly modified and constrained throughout the project's life cycle on the client's instructions, the interior designer, and input from other experts [1]. It is materialised in the assessment, documentation, and layout of constructions. In Structural Engineering Companies (SECs), connections between experts inside and outside the organisation and workflows often lead to poor information distribution, insufficient communication pathways, revamps, and frequent modifications, among other conditions that reduce productivity [2]. The advancement of a thorough virtual prototype for the various stages of a project's life cycle has been made possible by Building Information Modelling (BIM), among the most significant and appealing modifications in the architecture, engineering, and construction (AEC) companies. BIM denotes a conceptual shift in the project's gestation and conception. BIM enables unification in the typically fragmented AEC industry, enhancing harmony and collaboration while reaching more significant levels of effectiveness [1]. These approaches and technologies have been currently allowing the administration and analysis of the produced data for extensive, complicated engineering activities. Considering that structural behaviour must be meticulously examined while adhering to several regulatory requirements, not to forget professional practices, the structural layout phase constitutes among the most challenging and dynamic activities in the project's life cycle. Because of this importance, the structural layout process is crucial in creating the BIM prototype [3]. In the built surroundings, BIM acceptance and use have been gradually rising. Maintaining a correct balance between the project administration triangles of scope (features & quality), expense, and time has become among the main drivers behind BIM adoption because it addresses the most essential issues of users in the AEC sector.

BIM has been described as “a digital illustration of physiological and functional considerations of a facility” by the National Building Information Model Standard Project Committee. A BIM has also been described as “a shared understanding resource for data about a building developing a trustworthy context for choices during its lifecycle; described as occurring from oldest conceptualization to destruction [4].” There isn't general agreement on what the word “BIM” truly entails because of the different methods taken to describe its meanings. These can be process-oriented methods, methodologies, or technologies. This research automatically views BIM as a technique in Succar's description: “a set of conversing policies, procedures, and techniques producing a technique to handle the crucial building layout and project information in digital sequence throughout the structure's lifecycle.” This seems to be because of the multidimensional and systemic essence of BIM. Therefore, implementing BIM requires a series of tasks that enable adopting transformational ideas and technologies (either revolutionary or incremental) throughout an organisation [5]. All parties participating in the BIM execution may become more productive, which is advantageous for the entire building supply chain [6]. While BIM can offer a wide range of features and a commercial edge, its total capacity is still under-utilised. This seems to be partially a result of the anticipated challenges with organizational-level BIM integration. Although most managers' primary concern with BIM adoption seems to be economic productivity, they have been unable to develop a complete plan to optimize the advantages of BIM deployment [7]. BIM adoption entails distinct expenses and frequently requires significant resources to overcome implementation challenges. Because of these challenges, a firm may accept BIM ideas and tools but mayn't be capable to completely realise their advantages, leading in capital waste and a regression to more conventional ways.

Implementing BIM seems dynamic, and several vital aspects may alter over time. However, the characteristics of the changing environment and AEC firms' capacity to handle BIM uptake and execution within enterprise contexts have received microscopic study. Further obscuring the BIM adoption seems to be the absence of specific BIM implementation standards. A BIM implementation paradigm has been created to give the actual implementation procedure a firmer conceptual foundation. This will make it easier for AEC firms to incorporate and put into practice stronger procedures and transition management techniques. A combination of the current BIM, innovative management, and data technology platforms literature identifies the key enablers for effective BIM deployment. Additionally, complicated geometric building layouts are a common feature of contemporary architectural projects, complicating the structure's structural analysis [8]. Despite the preceding, there has still been no technique for data transfer at the architectural design phase that is universally acknowledged, making it the weakest connection in BIM prototype processes [9]. Since the effectiveness of BIM relies largely on the effective information exchange created by many professions, it becomes crucial to overcome this latter obstacle and effectively integrate structural area operations into the typical project's work chain [10]. The importance of using BIM in 3D modeling stems from the early detection of problems such as structural interference with MEP services, which allows for avoiding design errors and addressing problems in the design stage, which saves time and effort. It also defines project participants' tasks and responsibilities, improving the product's quality and completion. With the development of a technique for BIM execution in structural engineering firms (SEF), such as processes, interrelations, and workflows; suggestions for computer programs and communications systems; and other factors required for the achievement, the main objective of this paper is to provide an option to the current difficulties within conventional industry norms.

### Implementing BIM as a firm process modification

Implementing BIM could help AEC companies establish new products and organizational processes to provide end consumers with the most value. BIM presents a singular opportunity for AEC organisations to either modify their current procedures or develop new ones because it is a business process transformation enabler. A company must be repositioned to adopt BIM, and its organizational methods and procedures must be transformed, not only the software tools used. A group of synchronized operations or logically connected actions carried out to provide value to end clients or accomplish other operational goals is referred to as a business operation. Analysing and continuously improving an organization's efforts and operations is essential for business processes' effective management. To provide organisations with the chance to benefit from BIM adoption fully, there requires more attention paid to business process facilitators besides enabling technologies. A socio-technical systemic strategy is necessary for an effective BIM implementation, which entails managing people, changing the culture, and making significant adjustments to business operations and workflows [11].

### Research background

#### BIM concepts and definition

Before the project is created, BIM provides an opportunity to correct design flaws and/or execute adjustments by reproducing a structure's structural and functional aspects. Due to its untapped ability and capacity to enhance efficiency in the AEC owner operated (AECO) market, BIM has drawn much interest from academics and businesses. The Associated General Contractors of America (AGC) describes BIM as “an info-rich object-oriented, smart and generalized linear digital depiction of the venue, from which beliefs and statistics suitable to numerous users' requirements can be retrieved and evaluated to produce data that could be employed to create decisions and enhance the imparting the facility procedure,” even though there are numerous other descriptions for BIM [12]. Meanwhile, BIM encompasses more than merely the digital depiction; rather, it signifies a fundamental change in how buildings are delivered. The present industry tendencies toward fully automated project operations depend on this process transformation, also referred to as “combined practise” or “incorporated project execution.” [13].

#### Implementing BIM's critical success factors (CSF)

Several CSFs for applying BIM in the AECO sector emerged over the past ten years, particularly in improving cooperation among cost estimation, project stakeholders, and quantities take off, as well as the interaction between various project partners (using a shared data context). Azhar et al. [14] claim that since BIM has been capable of storing extensive geometrical or semantics information, a common data environment (CDE) might lessen errors caused by inconsistent and disorganised project documentation. Additionally, the thoroughness of information interchange enhances the construction administration lifecycle and advances sustainable building architecture. Given the tangible advantages offered, facilities executives employ BIM during the operation and maintenance (O&M) phases of a structure's life cycle, such as: maintaining guarantee and service data; quality assurance; evaluating and monitoring the governance of space and energy; urgent management; and/or retrofit schedule. Implementing BIM also makes it easier to coordinate the planning of layout and operational activities. Building stakeholders can more specifically use 4D modelling to see the constructability, scheduling, and scheduling of a suggested construction technique [15].

### Related works

Some of the recent literatures related to the BIM are described as follows:

In the last few years, BIM has become increasingly popular as it has been adopted and used in building design across the globe. Chan et al. [16] aimed to determine and evaluate the perceived advantages of and obstacles to BIM adoption in the Hong Kong building sector. This research used a systematic scientific questionnaire assessment as a quantitative investigation strategy. The respondents' groupings perceptions were also compared and analysed. The main obstacles to BIM acceptance seem to be the absence of BIM industrial norms in Hong Kong, insufficient organisational assistance, and framework for BIM implementation, and building stakeholders' innate reluctance to change. The main advantages, meanwhile, involve improved cost estimation and regulation, effective scheduling and management of building projects, and an enhancement in layout and project integrity. Policymakers, local governments, construction companies, and other important stakeholders have been given helpful and informative advice on expanding BIM adoption in infrastructure projects and supporting building design as a whole. However, a case study investigation was not performed.

It is well known that BIM may make prefabricated construction easier to deliver. However, there are many obstacles in the way of BIM in reality. Various studies and literature have thoroughly examined these hurdles, but 2 study questions are still open. What particular obstacles must BIM users in China's prefabricated building overcome first? How would these impediments connect, secondly? These were the two issues Tan et al. [17] have resolved. A questionnaire inquiry and a 2-round literature study were conducted to identify the twelve major obstacles that the Chinese experience with using BIM to prefabricated building faces. Interpretive Structural Modeling (ISM) has also been utilised to determine how these barriers interacted. This research adds to the corpus of information by identifying key obstacles to BIM adoption in China's prefabricated buildings and developing a matching three-level plan to make adoption more likely. The results of this research can thus serve as a useful guide for future studies that looks for organizational and technical improvements to BIM deployment in China's prefabricated building. However, the hypothetical model's validity was not assessed, which requires the analytical model's integration.

Due to new technology, the construction sector has been currently going through a digital transition. New organisational structures have been therefore required. Collaboration with BIM seems to be difficult and presents difficulties for project administration. In BIM-based initiatives, multidisciplinary players frequently share digital information, which triggers complex inter-organizational interactions. Through boundaries and theoretical lenses, Papadonikolaki et al. [18] performed a study that provides insights on cooperation with BIM. The interaction between architecture and agency of cooperation has been examined through the analysis of 2 BIM-based cooperation projects in the Netherlands. Several groups of practice understood the numerous BIM components as boundary items in several ways, which led to poor cooperation and bad communications. The research refutes the conventional understanding of BIM as a software product and demonstrates that this understanding only substantially promotes cooperation.

Furthermore, the deployment of digital technologies can be completely supported by a structuration perspective (supported by communications, negotiation, dispute management, and cooperation) as contrasted to a structural perspective of cooperation (for example, BIM as software). The research ends with recommendations for assisting team structure through the BIM-based management cooperation in initiatives and going beyond the purely architectural and technical methods that now dominate the area. Moreover, the empirical data was not validated well in this research.

To investigate and evaluate the CSFs that could enhance the incorporation of BIM and environmental practices in building projects, Fan [19] have set out to study and evaluate the CSFs. Using a 2-round Delphi survey, the Delphi survey approach has been used to get the opinions of specialists on the 30 detected CSFs. Statistical empirical and confirmatory analysis tools have examined the professional panel's answers. People-centric, data-centric and technology-centric innovations in building design seem to be the main forces cited in the research. On the premise of a comprehensive examination of the research groups, significant conclusions have been drawn. The study's conclusions have given local governments, legislators, and project stakeholders useful guidance for accelerating the implementation of green BIM efforts. The research has added to the corpus of information regarding smart urbanisation and practical experience in building design, as well as effective suggestions for boosting the adoption of BIM and sustainable practices in the construction sector. However, the case study was not investigated by this research. BIM was just introduced in Malaysia and was growing in acceptance. The adoption and implementation of BIM in the building sector in Malaysia have also received the majority of attention in the literature, but its efficacy was not studied. Kong et al. [20] have examined how BIM has affected the Malaysian building sector. Interviews with professionals with past experience in the building sector and BIM have been done using a qualitative method. The determining factors were identified and categorised using the data from the questionnaires. It has been discovered that the lack of modelling guidelines and the continuous requests for design modifications make BIM adoption in the Malaysian building sector inefficient concerning cost and time. The findings also suggest that Malaysian BIM seems to have the ability to be as efficient as that in most industrialised nations, provided that the key issues raised are resolved.

Because of the absence of knowledge on methods and components, there have been many ambiguities in the early stages of architectural design. As a result, architects and designers cannot measure the ecological effects of structures to assess their projects' earlier environmental efficiency. Rezaei et al. [21] life cycle analysis (LCA) and BIM have been used during the preliminary and comprehensive phases of architectural design. The technique has been used on residences in Québec, Canada. Revit can be used for BIM, and openLCA has been used for LCA. A useful database has been created to provide the Revit outcomes as the LCA models' proper inputs. It often covers all structural components, layers, and potential materials in Québec residential structures. Information for every material's life cycle inventory (LCI) has been sourced from the ecoinvent dataset. Each material has been given a probability function to handle information ambiguity during the earliest design phase. All material kinds and quantities have been defined in the BIM file utilised during the final construction stage of the LCA research. The building phases and assemblies' environmental effects were computed to identify the best construction assembly choices from an ecological standpoint. This process could help to build developers in the environmental evaluations of their models, making it feasible to choose more environmentally friendly materials for every arrangement and thereby lessen the building's ecological impact. Moreover, the environmental impacts were not assessed completely.

Building environmental effectiveness evaluations may be facilitated with the help of digital solutions built on BIM. Furthermore, several tools have been created that leverage a BIM framework for automated quantity take-off as the foundation for LCA. The first use of a BIM-LCA tool to assess the embedded global warming potential (GWP) during the real building's entire design procedure has been detailed by Hollberg et al. [22]. The BIM framework's 34 phases have been examined once every week. The findings demonstrated that the embedded GWP during the designing phase was double that of the finished building. Along with other factors, the architects' strategy of employing placeholder elements later modified can be largely blamed for these modifications. As a result, the embedded GWP has been vastly overstated, and an environmental evaluation predicated on BIM throughout the design phase may be deceptive and harmful. Three options for the current automated quantity take-off have been discussed for future improvements. Furthermore, this method necessitates the creation of a sizable library of as-built BIM designs that contain the necessary data for LCA.

Olawumi and Chan [23] created a BIM-project information management framework (BIM-PIMF) and an accompanying evaluation system for building projects to improve the project information's functional management. The article's research strategy comprises an interpretive case study methodology and case study data from 4 BIM building projects. The research identifies and develops the BIM-PIMF model's 3 sub-criteria: the essential metrics for an effective BIM installation on building construction activities, the BIM procedure level variables, and the BIM products level variables. On a 5-point quantitative scale, these characteristics have been semantically related to the creation of the BIM-PIMF architecture. The BIM-PIMF concept creation and associated analytical scoring method have been one of the article's outcomes. The study's conclusions enhanced project personnel's technical competency while facilitating information channels and simplifying the incorporation of technological advancements in building projects. The research brought to light a variety of useful suggestions and tactics to improve the use of BIM across the lifecycle of a project. Policymakers and government agencies could use the model to determine which construction businesses should receive subsidies by measuring how much BIM is used in projects. However, needs more enhancements in project data functional management.

For the field of AEC, BIM presents some options. Since the human element have heavily influenced the acceptance of this smart 3D model-based approach, its effective execution must guarantee the key stakeholders' preparedness for change. Britel and Cherkaoui [24] have analysed the modified readiness for the deployment of BIM inside a construction project administration organisation in Morocco. The suggested assessment system bases its analysis on linguistic parameters and the Fuzzy Analytic Hierarchy Procedure. As a result, considering the participants' confusion, the suggested strategy aids in creating an enhancement roadmap in addition to revealing the firm's readiness maturity stage. However, this model requires more enhancements in the management of construction projects. A new method in the building sector was BIM. Although it is thought to be a successful strategy for creating green buildings, its usefulness from a lifecycle viewpoint has not been fully examined. Wen et al. [25] used a Convolutional Neural Network (CNN) technique to examine the utility of BIM implementation in various green building stages to close this research gap. First, a methodology for evaluation was created while keeping the balance between approximation accuracy and data volume in mind. The constructed model's validity has been confirmed from both intellectual and empirical angles. Ultimately, the suggested methodology has been used to evaluate the BIM effectiveness. Results indicated that the project under test had an entire Likert-scale value of 4, with a mean relative inaccuracy < 1 %. The BIM's implementation value indicated a falling order across the tested project's lifecycle from a value-based viewpoint. Additionally, it has been discovered that the social significance received the lowest score while the operational value received the greatest. The outcome of this research can assist decision-makers in identifying the shortcomings of BIM execution during the creation of green buildings. Moreover, this research has not analysed the building's reuse and recycling phase.

## Method details

**Fig. 1:**
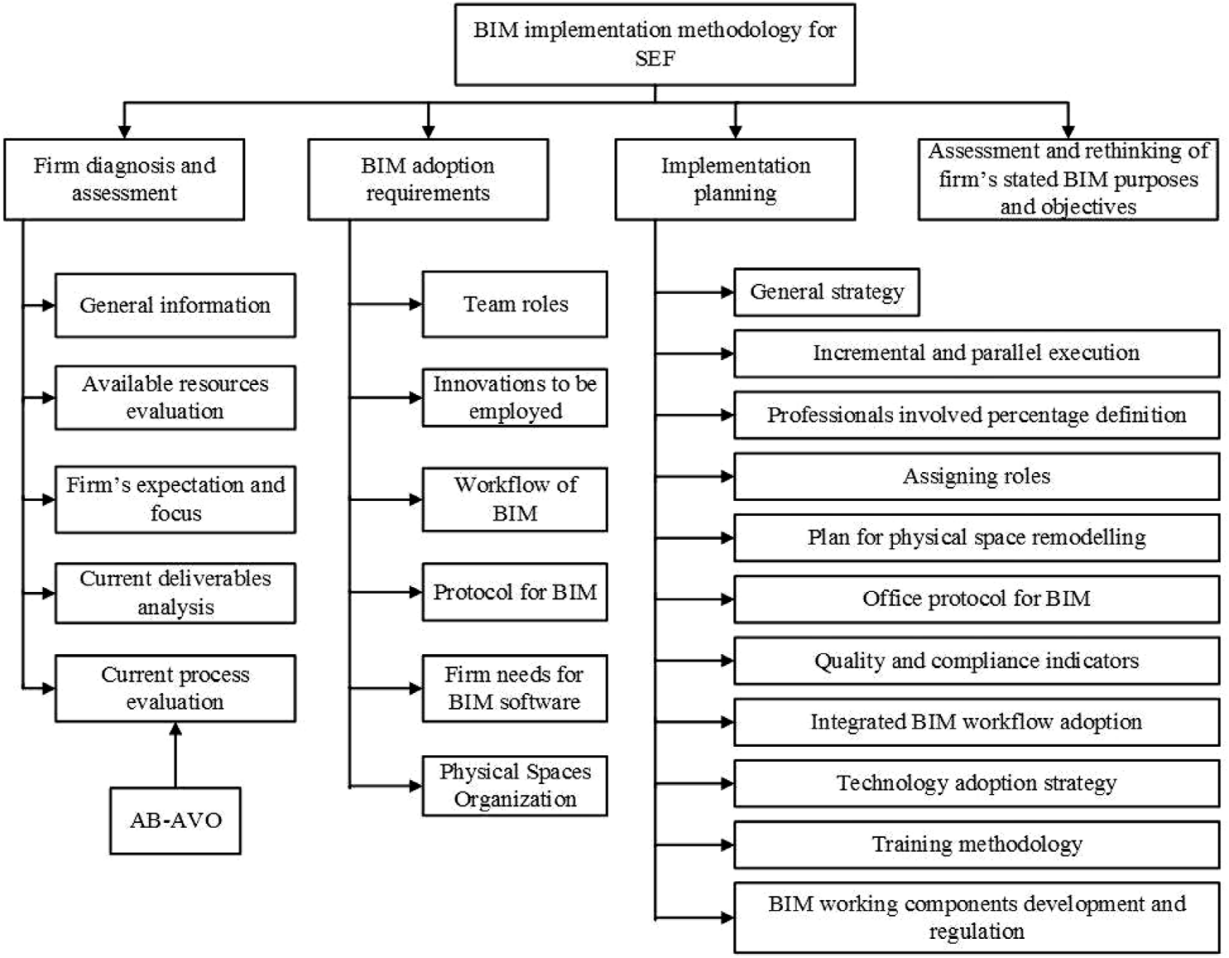
BIM execution approach phases (adopted by authors).

The phases of the BIM execution approach for SEF are depicted in Fig. 1. While extending and adopting the same for SEC, the approach preserves the manual implementation concepts from renowned authors, analytical guidelines, patterns, and guidelines of the “BIM Handbook” as well as the “Project Execution Planning guide.” Real execution needs are defined, resources and procedures are objectively evaluated, clear and adaptable procedures for firm requirements are established, and expenses are maximally minimized. As per perspectives evidenced in numerous research studies, the prerequisites for an execution approach inevitably include recognising the purposes, anticipations, and strategies that a given firm wants to accomplish when implementing the BIM technique; recognising roles, groups, and functional systems; planning progressive scales and execution and training speeds; and recognising the management and staff alignment, together with a thorough strategic plan. The prototype in this paper includes additional elements in addition to the suggestions mentioned earlier to produce a more thorough and adaptable implementation strategy. These additional elements are broken down into six main areas: firm evaluation, firm's BIM purpose reformulation, needs for adopting BIM, identification of the “execution gap,” techniques and planning for execution, and evaluation and monitoring as shown the Fig. 1 below.

BIM implementation methodology for structural engineering firms approach phases is shown in detail in Fig. 1. It can be realized that software and Hardware should be listed in the firm's technical resource assessment, along with any virtualized tools, platforms, or other tools that have been employed. Therefore, these media items can be divided into at least three broad classifications: Hardware (model, brand, processor, RAM, video card, video adapter, and hard drive); virtual and software platforms (local provider, name, developer, type and expense of licenses, summary of use); as well as “cloud” and local servers (capacity, model, brand, and network summary). The physical work within the business must permit the fluid communication required for BIM execution between project team participants. This is why, during the BIM implementation, the organization must submit its plans for the physical facilities that are currently in use, outlining the sites of buildings, systems, furnishings, and individuals to comprehend conflicts involving staff communication in the office and suggest restructurings tailored to the current situation. BIM the program works on an integrated team, and as mentioned previously, defining responsibilities and coordinating the distribution of information allows companies to work better and produce a highly efficient product.

### Firm assessment and diagnosis

It is important to comprehend how the organisation functions, its assets, and its objectives and future predictions to refocus firm activities utilising the BIM approach. By doing this, the execution will align with the company's goals, vision, and purpose, make the best use of its resources, and produce the most appropriate plan. Management employees must receive BIM training from the moment they connect with the organisation to familiarise them with the technique and demonstrate its possibilities. The following step is to generate all the relevant information elements listed below to analyse the firm's activities and identify its requirements thoroughly.

#### General information

It is necessary to gather general data about the business to identify it and choose appropriate administration in the future. The following information has been required: the firm's name, professional contact information, contact address, the type and number of professionals, organisational chart, working hours, and training session schedules.

#### Available resources evaluation

Three categories—human resources, technical resources, and office and physical space furnishings—are used to analyse the accessible resources. To detect previously allocated resources and correlate some operational expenses, it has been required to understand renovation, investment, or expansion intentions for every of these. The obtainable human resources have been assessed to learn more about their capacities and competencies, including technical and interpersonal skills like technical competencies (TC), mentality and willingness to change (MWC), personal and collaborative work skills (PCWS), and alignment with the firm's development and vision (AVDC). The self-assigned ratings from every professional (Pp) must be compared to the assessment of their direct supervisor (Ps), at the evaluator's option, to ensure high standards of honesty throughout consultations. The talents and abilities listed in Table 1 must be discussed with staff members of the firm. Based on the business context, certain items for particular computer programmes must be incorporated or eliminated. While not comprehensive, the list contains the most commonly discovered programmes in the offices surveyed.

**Table 1:** Measurement instruments for human resources.

Item	Sl.no	Competencies
PCWS	1	Creativity
	2	Discipline sense
	3	Leadership
	4	Persuade ability
	5	Negotiation skills
	6	Problem solving knowledge
	7	Collaborative work
	8	Communication skills
	9	Management control knowledge
	10	Conflict management

AVDC	11	Alignment with firm's mission
	12	Organizational sensitivity
	13	Alignment with firm vision
	14	Commitment to firm

MWC	15	Self-study capability
	16	Readiness for additional studies and learning
	17	Quality orientation
	18	Willingness to adapt

TC	19	SAFE mastery
	20	Advance steel mastery
	21	Mastery archicad
	22	Tekla structures mastery
	23	SAP2000 mastery
	24	AutoCAD-2D mastery
	25	Programming program's mastery (MatLab, other)
	26	Structural design's mastery standards
	27	BIM methodology's mastery
	28	Naviswork's mastery
	29	Mastery ETABS
	30	AutoCAD-3D mastery
	31	Revit MEP mastery
	32	Structural robot mastery
	33	Advanced concrete mastery
	34	Other BIM project's mastery
	35	Other structure and master programs
	36	Revit structure mastery
	37	Working in the cloud mastery
	38	EXCEL's advanced mastery
	39	Revit architecture mastery
	40	Plan describing standards mastery

#### Firm's expectations and focus

The implementation strategy should align with the firm's mission, vision, and goals for using BIM. Three perspectives should be described as a result: (1) The firm's vision; (2) The projects and target market created; and (3) The goal of implementing BIM. Considering the firm's descriptions of how it was created, how it had behaved, and how it projected itself into the perspective has been necessary to recognise its goal and vision. Respondents need to be prepared to explain how BIM would help achieve these organizational goals. Inability to communicate timely needs and allowances for different contractors in harmony with BIM outcomes, the organisation should be clear about its target market as well as the size, kind, and project's estimated duration it is producing. The organisation must also specify the goals behind its BIM adoption. Lower prices, better project quality, quicker completion, entry into new sectors, or regulatory restrictions are some examples. The company accomplishes these aims as reflected in expectations of tangible outcomes (tasks and dates).

#### Current deliverables analysis

The firm should report current deliveries. Understanding the features of organisational deliverables has been necessary because the result of implementing BIM must be in line with present indicators. The “Traditional Design and Drafting Practices Manual”, which describes the development of the plan generated under conventional work techniques and standardises work done inside the SEF, should contain all deliverables an organisation currently possesses. The firm's goal has to make three features clear: (1) the minimal regulatory approach necessary; (2) SEF standards above regulatory needs; and (3) formed checkpoints for data verification at all project advancement stages to stop the errors spread and to strive prompt correction. Its verification shouldn't be difficult because many businesses already have this paperwork for office requirements.

#### Current process evaluation using hybrid African Buffalo and African Vulture Optimization (AB-AVO)

Current workflow and procedures, programmes utilised in each activity, and existing challenges are the three criteria used to evaluate current processes (and their constituent parts) within the firm. All kinds of resources and deliverables must have established processes and workflow inside the firm. Firms in the field don't typically describe processes explicitly; nevertheless, experts typically have a defined definition. The evaluation procedure is subsequently transformed into a workflow pattern by the assessor. Any applications that create or assist the work must be identified for every workflow's specified activities. This aids in identifying the firm's present issues. African buffalos' three principal characteristics are extensive memory capacity, cooperative cum communicative ability in both good and bad times, and extreme intellect gives origin to a democratic character. There are two sounds: ‘*maaa*’ and ‘*waaa*’, which are indicated by $P_{k}$ and$\;S_{k}$, respectively. The buffalo's movement is determined by Eq. (1).

(1) $$P_{k}+1=P_{k}+V_{1}\left(d_{r}-S_{k}\right)+V_{2}\left(d_{u}.k-S_{k}\right)$$

Here, the exploration is denoted as$\;S_{k}$,$\;P_{k}$ is the exploitation moves, k represents the iteration, and the learning factors are represented as $V_{1}$ and$\;V_{2}$, the herd's best fitness is represented as $d_{r}$ and $d_{u}.k$ is buffalo's best in each iteration. The current process and workflow are updated by Eq. (2).

(2) $$S_{k}+1=\left(S_{k}+P_{k}\right)\;/\;\pm 0.5$$

Up to meet the required criteria, the process is continued. Moreover, the African vulture's four principal phases are the best vulture determination from any group, vulture's starvation rate, exploration, and exploitation. After data collection, all solutions fitness is computed; the entire data is computed in the iteration for each data. It is computed by Eq. (3).

(3) $$S_{l}=\left\{ \begin{array}{c} BestVulture_{1}ify_{l}=P_{1}\\ \begin{array}{c} \ldots .\\ BestVulture_{n}ify_{l}=P_{n} \end{array} \end{array}\right.$$

where, $S_{l}$ indicates one of the finest vultures. The probability of selecting the chosen parameters to move the other parameters towards the best solution in every group is determined by Eq. (3). Here, $P_{1}$ and $P_{n}$ are the parameters. The parameters should be evaluated before the searching process, with values ranging from 0 to 1, and the total of both variables equal 1. The probability of identifying existing challenges is identified by employing the Roulette wheel to select the finest solution for every group by Eq. (4).

(4) $$y_{l}=R_{l}\;/\;\sum_{l=1}^{n}R_{l}$$

The mathematical modelling of the vulture's behaviour is done by Eq. (5), which is represented below:

(5) $$p=w\times \left\{sin\left(1.57\times i_{i}\;/\;maxi\right)+cos\left(1.57\times i_{i}\;/\;maxi\right)-1\right\}$$

(6) $$R=\left(1-\frac{i_{i}}{maxi}\right)\times u\times \left(2.rand_{1}+1\right)+p$$

where R indicates that the programs utilized in every activity, the current number of iterations is represented as$\;i_{i}$, the total iteration numbers is denoted as maxi, the random number is indicated as u, which is +1 and -1 that changes every iteration, w is the random number ranges between -2 and +2, the random value of $rand_{1}$ is 0 and 1. When the value of w is greater than zero, then the parameters are satiated. Each vulture looks for its satiation in the environment randomly. This process was represented in Eq. (7).

(7) $$y\left(l+1\right)=\left\{ \begin{array}{c} \left(\left(S_{l}-Q_{l}\right)\times R\right)ify_{1}\geq rand_{y_{1}}\\ \left(S_{l}\right)-R+rand_{2}.\left(\left(U_{b}-L_{b}\right)\times rand_{3}+L_{b}\right)ify_{1}<rand_{y_{1}} \end{array}\right.$$

(8) $$Q_{l}=\left|D\times S_{l}-y_{l}\right|$$

where, y(l+1) denotes the current process in the next iteration, D denotes the vultures movement, and it is utilized as a coefficient vector, which enhances the random motion. $L_{b}$ and $U_{b}$ represents the variables lower and upper bound. Moreover, a rotating flight strategy has been carried out if the random number is smaller than $y_{2}$ parameter; the process is shown in Eq. (9).

(9) $$y\left(l+1\right)=\left\{ \begin{array}{c} Q_{l}\times \left(R+rand_{4}\right)-h\left(t\right)ify_{2}\geq rand_{y_{2}}\\ \left(S_{k}+1\right)ify_{2}<rand_{y_{2}} \end{array}\right.$$

(10) $$h\left(t\right)=S_{l}-y_{l}$$

where, $rand_{4}$ indicates the random number between 0 and 1, which is employed to enhance the random coefficient.

### Rethinking and assessing of firm's stated BIM objectives and purpose

In certain circumstances, rather than utilising BIM to its full potential, the organization's stated aims for using it could result from incomplete or erroneous knowledge. In light of this, the objectives to be reached through BIM must be reviewed to maximise resource utilisation for expenditures or to establish concrete targets on the aspirations created once firm goals were identified and positioned within the characteristic's paradigm (resources, sizes, etc.). The accomplishment of goals should be spread out all through the medium, short, and long durations.

### Requirements for BIM adoption

The implementation strategy takes into account the significant contributions from existing organisational traits and resources to identify all the needs needed for a SEF to operate with BIM.

#### Team roles

The growth of structural designing and computation under the BIM technique requires adapting existing generic BIM roles because the execution plan in this study concentrates on SEF. It is crucial to remember that BIM roles offer duties and tasks to various team members; they have not always been associated with positions or specializations, and they can also be created by many people or permit one person to perform multiple roles. The present SEF BIM plan takes into account the following five roles, which are described as follows:

• ***BIM leader*:** Accountable for leading the firm's BIM deployment, establishing guidelines, and directing the BIM execution plan (BEP). Moreover, thorough BIM methodology knowledge is required.
• ***BIM reviewer*:** In charge of ensuring that the modelling was accurate following firm protocols, technological standards, and normative considerations.
• ***BIM coordinator*:** Model evaluation and collaboration are the responsibilities of the firm's BIM procedure articulator. Provides a focal point for communication between various modellers and specializations; adheres to the BEP and is fully conversant with BIM principles, requirements, and regulations.
➢ ***BIM modeller*:** Responsible for creating BIM representations, including information on the components and 3D visuals. Must thoroughly understand the underlying computational techniques and the discipline being modelled.
➢ ***BIM project engineer*:** Professionals with experience in modelling, analysis, and architectural design but who have also learned to produce these tasks partially or entirely using BIM techniques and computational technologies.

In an attempt to objectively determine the needs for the various qualities and capacities of professionals taking a specific BIM function, the positions' skills and competencies have also been adjusted to numerical variables. Moreover, various BIM roles require specific professional abilities and competencies are shown in Table 2. The skills quantitative measure scale ranges from 1 to 5, where 5 indicates a higher competency level and 1 indicates less competency level.

**Table 2:** BIM roles specific professional abilities and competencies under different skills.

Sl.no	Skills	Leader	Reviewer	Coordinator	Modeller	Project engineer
1	Training	4	4	4	4	3
2	Management control	5	3	3	2	3
3	Creativity	5	3	3	4	4
4	Vision	5	3	3	2	3
5	Persuasion	5	3	1	4	4
6	Communication skills	5	3	4	1	3
7	Discipline sense	5	4	4	3	4
8	Willingness to adapt	4	3	4	3	4
9	BIM methodology	5	3	3	3	2
10	BEP	5	2	3	2	2
11	Structural layout and computation	2	1	1	1	5
12	BIM structural computation program usage	2	1	1	1	5
13	BIM coordination initiative usage	2	5	5	3	2
14	Leadership	5	2	3	1	3
15	Collaborative work	5	4	5	3	4
16	Problem assessment	5	3	5	2	5
17	Firm sensitivity	5	2	3	2	3
18	Conflict management	5	3	4	1	2
19	Negotiation skills	5	2	3	1	2
20	Quality orientation	5	5	5	5	5
21	Industry requirements and challenges	4	1	1	1	2
22	Implementation technique of BIM	5	2	2	2	2
23	Structural computation program usage	2	1	1	1	5
24	BIM norms and standards	5	4	4	4	3
25	BIM interaction platform usage	5	4	5	4	5

#### Innovations to be employed

The productivity of the workflow suggested by the BIM approach depends on the software interoperability used to operate in BIM settings. Although industry foundation classes (IFC) appear to be a common language to link up many software programmes in BIM surroundings, the technique has still not been fully addressed; at the moment, the only way to properly connect frameworks from different forums has been to use native programmes, i.e., those from the identical provider or with alliance providers. Additionally, given the range of possibilities available on the market, selecting the precise instrument that achieves the desired goals is essential, taking into account its ease of use and compatibility.

BIM software demands more processing power. The suggestions in Table 3 follow guidelines established by agreement among programme brands and knowledgeable user perspectives. The project's size to be modelled directly affects the necessary hardware capacities. Thus, these have been stated to cut down on equipment expenses that could not be utilised to their fullest capability in the medium or short terms. Operating system, hard drive, processor, RAM, and visual card are the five evaluation criteria that are specified. Table 3 lists common hardware needs and suggests solutions based on project size. Medium-sized and big buildings were referred to as “Type I” projects (e.g., medium-sized offices), and huge, complex works were referred to as “Type II” projects (e.g., large airports, hospitals, etc.).

**Table 3:** 2018 BIM software's hardware suggestion.

Sl.no	Item	Project type	Recommendation
1	Hard disk	1	Conventional 1TB HDD disk + 128GB SSD
		2	500GB SSD
2	Graphics/video card	1	Dedicated NVIDIA GC
		2	Dedicated NVIDIA GC
3	Operating system	1	64bits, Windows®10 Microsoft®
		2	Windows®10, 64bits Microsoft®
4	RAM	1	16GB
		2	32GB
5	Processor	1	Core I7-Intel®
		2	Core Xeon-Intel®

Note: SSD-Solid State Disks; GC-Graphic card

All computers in the office employees must be connected to a network because the linkage of files, processes, models and professionals forms the BIM basis. For instance, working with Microsoft's “Windows Server” system has many security benefits, including support for cloud services. Additionally, simulations must be able to be visualised and coordinated from any physical place. To do this, it is advised to make cloud computing platforms an advantage, such as A360 and BIMsight Key, amongst many others, to enable online collaboration with other project participants. In the future, computerized assistance will be required to handle projects that involve many data. It'll be important to govern the projects' big data by optimising it.

### Physical spaces organization

In a cooperative setting like BIM, the physical location distribution greatly impacts how experts improve their tasks. It is vital to redesign the workplaces within the business to generate more and better relationships. In 10 SEF in Dubai, field investigations have been performed, and it was noted that the engineers and modellers have always been divided. According to experts, there have been communication issues between engineers and modellers, primarily due to how the roles are divided and the need to go to different locations to consult on projects. The physical configuration known as “3 pairs” has been suggested based on investigations done in the environment at various local businesses. Three different sorts of professional pairs are used in this setup: modeller-modeller, engineer-modeller, and engineer-engineer.

Thus, engineers and modellers have been apt to interact directly, and engineers acting as modellers (designer draughtsmen) were also possible to give feedback to one another, etc.; in other words, each may straight consult their co-worker next to them about any hypothetical or technical doubts related to their line of work. It is advised to place more seasoned experts at the extremities of the “chains,” wherein there has been only one expert left unpaired, as they will confer with their peers less frequently and take up less time ultimately. Professional BIM modellers and coordinators must operate simultaneously in an incorporated collaborative workspace connected to the incorporation of other specialisations (apart from the engineering-designer computation function) in what was referred to as the “extreme collaboration environment.” The use of a large space was beneficial to draw the proprietor and the other specializations together and achieve an incorporated collaborative approach. Professionals may operate from their laptops and observe the main layout. All project participants, along with the constructors and architects, can meet and make decisions in the extreme collaboration environment to uncover mistakes or better approaches to develop the prototypes. The various project participants can physically collaborate in this space while viewing real-time visuals of how choices have been carried out (in 3D).

#### Workflow of BIM

The workflow for the presented BIM approach enables smooth document creation and interaction operations and model revision, which ultimately saves time. Predicated on expert communications in a central prototype, this workflow seems to be a generic BIM flow adaptation suggested in the project implementation planning guide. The BIM framework for a provided SEF (3Ds Max, for instance) will encompass volumetric methods, other architectures or reinforcement steel as suitable, and thorough layouts and illustrations. As a result, all the simulations might be “superimposed” to highlight disputes and enhance engagement. Additionally, the process suggests holding coordination meetings with all the pros to progress standards and settle on modifications.

#### Protocol for BIM

The task that architectural design firms conduct is defined by their guidelines of rules and processes, which have been predicated on the norms and national architectural regulations. Currently, organisations are directed by 2D design and drawing practice guidelines to standardise their layout and detailed outcomes predicated on the recorded CAD-2D drawings (complemented by three-dimensional assessment designs). A corresponding document called the “BIM Protocol” needs to be developed for documentation using the BIM paradigm to perform in BIM. This will include the minimal regulatory approach necessary, standards formed by the SEF (in addition to the regulatory needs for modelling, following the goals described by BIM), and regulate points for validating data at all stages of project growth in an attempt to stop errors spread and pursue their prompt correction. To standardise model creation on BIM interfaces, provide work systems, specify channels, and link models and experts, this must be in line with the BEP. It'll be a fluid document that may be modified in response to legislative demands and technology advancements. The BIM protocol recommendations with their description are described as follows:

• ***Responsibilities:*** The following duties were recognized, developed, and designated: staff in charge, commitments, execution, oversight, and adherence.
• ***General terms, features, and definitions:*** As per the firm's requirements, general objectives should be plain. Define, among other things, bullets, work and scales units, formats, updates and changes, sizing and dimensions.
• ***Plans contents:*** The final designs, which may be created in conjunction with the BIM prototype, must adhere to conventional SEF criteria so that their contents and features do not differ from those listed in the firm's Traditional Design and Drafting Practices Manual.
• ***BIM considerations and definitions:*** The team needs to be placed within the new work methods context in light of the preceding factors: prototype transcendence (whereby the modelling must've been helpful "upstream" during future phases), LOD (levels of detail) and LOI (levels of information) in the methods, interconnectivity and IFC, fluid interaction significance, BIM calculation tools, interrelated work "in the cloud," amongst many others.
• ***Project creation and workflow:*** It is necessary to list all project stages, input files, and outcomes (background, computation report, models, and documentation). There are specific workflows between external and internal professionals.
• ***Basic modelling components:*** Considering how specific activities should be created in various BIM platforms, fundamental modelling concepts should be highlighted. Specific design citations, programme commands, parametric component attributes, and annotator kinds, among others, have been noted.
• ***BIM modelling suggestions and strategies:*** Explain suggestions like conceptual model framing (dividing the prototype into specialities and levels to enhance workability), phases generation (organising distinct temporal project provinces, like construction and demolition), construction aspects (recreating the modelling as it is constructed in fact), factors for substrate take off, model cooperation, command in the project surroundings, subdivision by framework colours, and amenability.

The BIM Protocols must integrate all data repeated in the Conventional Design and Drafting Practices Manual (preferably using the conventional norm as a useful reference). To ensure that the activity has been appropriately standardised, particularly in the early phases of a project, office planning should state any deliverables with specific characteristics. Instances of the first designs should be provided after they are created to act as a reference for aspiring experts or to answer questions about how specific complex scenarios have been modelled. This protocol has been supposed to be adaptable and may be changed in the future if BIM aims continue to advance.

#### Firm needs for BIM software

According to the firm's “Conventional Design and Drafting Practices Manual,” it is presumed that the organisation already has a “design” for how it organizes and provides its goods (drawings). To ensure that office workers only utilise models from earlier developed templates, it has been required to create all of the layouts employed in the BIM software documentation. These materials must be accessible and given to the offices during the first execution stage for free.

### Implementation planning

The actions that'll be taken should be clear, specific, and detailed in the implementation plans. The supplied general rules must be customised to the specifics of the business. The BEP, which directs the effective creation of BIM initiatives, should include the points covered in this section. Below, key planning considerations have been covered, along with specifics on the goals and information each department is looking for. The methods indicated in the “BIM Handbook” and the patterns, and “Project Execution Planning Guide” served as the foundation for the following recommendations. They all proclaim their candidacy while considering the particular procedures created by the SEF and concentrating on its personnel, professions, and dynamics.

#### General strategy

The overall strategy should serve as the first source of inspiration for the workforce while keeping the firm's mission and vision front and centre. It should include a basic schedule outlining the steps necessary to accomplish the objectives, the BIM targets (explicitly labelled with the firm), and the execution plan's scope.

#### Incremental and parallel execution

In the firm, an incremental and parallel execution is now to be created. On the one side, the execution will be gradual; i. e., there'll be learning and different phases for execution (or usage), and after those have been successfully finished, the business will be able to go on to the next step. By doing so, quality will be guaranteed in accomplishing tiny goals, preventing errors from cascading further. However, the implementation must take place concurrently with conventional methods. Once control of that stage or target is accomplished, parallel activity can become a real chain's part; in other words, work that was previously performed in parallel (but has since been verified) can be included in actual process lines. As a result, professional processes have been constantly being added.

#### Professionals involved in percentage definition

It is important to identify the professionals who will receive BIM training. All employees in smaller and even mid-sized firms should complete BIM training and execution. However, a team of experts should be engaged in moderate-sized or larger businesses. In small businesses, handling, training, and taking into account the many professional duties of a smaller group of employees is much simpler. Considering that there aren't enough professionals to give each one a distinct assignment, this is particularly true. Conversely, large businesses typically form functional groups and areas for growth, so collaborating with all specialists at once would be overwhelming. Instead, the goal is to create a “BIM nexus” within the company, which will typically be the responsibility of further BIM knowledge development across the remainder of the firm and with any fresh employees who can benefit from formal training.

#### Assigning roles

By assessing current competencies found in the responsibilities of the workgroup and each professional's characteristics the office presently has, it is feasible to choose professionals who best fit the profiles needed for fresh BIM positions. Technical information should come after personal and cooperative work skills because it is simpler to teach technical information than soft competencies.

#### Plan for physical space remodelling

To adjust to the office size, a renovation plan must be provided, considering the physical space resources needed by the BIM process and the firm's existing physical condition. With the proprietors' cooperation, a recommended phased plan for site modifications and a switch to a different branch must be made. In addition, it is appealing to know its acquisitions and enlargement plan to direct the organisation with the necessary modifications. It'll be essential to restructure the group first and later (depending on the progressive execution progress and accessible resources), integrate the office design or produce the required physical adjustments.

#### Office protocol for BIM

To make it easier to grasp new standards, details, and required reassessments, the BIM Protocol must follow the same guidelines and sequence as the Conventional Design and Drafting Practices Manual, to the extent that it's been modified from it. The implementation firm will be in charge of creating the document, obtaining the necessary background data from the business, and offering suggestions and instances for how to use it. It is important to keep track of the various protocol upgrades that occur as BIM usage develops. If the firm doesn't have the same documentation or if it isn't being circulated and discussed by all teammates, it'll not be allowed to begin its function in BIM.

#### Quality and compliance indicators

The accomplishment of BIM aims and purposes within the organization is correlated with quality and compliance metrics. This means that the implementation development will be evaluated in terms of its capability, defined as the firm's aptitude for generating BIM characteristics and activities, and maturity, defined as the extent, depth, excellence, and repeatability of BIM characteristics and assistance. The metrics mentioned above contrast and categories the business within a specific range, which helps determine adherence with a competence profile demanded by a contractor, for instance, and facilitate standard markers of BIM methods advancement (and implications) in world terms (e.g., bidding bases).

#### Integrated BIM workflow adoption

The BIM criteria suggest an optimal workflow, although initially, a BIM's progressive introduction into the office must promote partial to full adherence to this movement. In light of this, the firm's claimed process should be modified to move toward incremental, partial substitute and, ultimately, the ideal BIM workflow. The transparency of goals reached will determine how quickly these changes happen.

#### Technology adoption strategy

A company must know current technical capabilities and features to determine its technological gap for working with BIM. In this case, it is also crucial to be aware of the strategy for purchasing and rejuvenating licenses and tools, as well as making the most of any resources that have already been budgeted for buying the platforms and tools required for the BIM technique's operations. The planning of this acquisition strategy should also take into account the traceability of stated goals. The implementation firm must handle installing licenses and setting up the corporate intranet. This will make it feasible to provide the services of license sales (via a distributor of programs who has been a significant partner) or to provide the organization with the option to decide. A technical team is also required to set up the hardware and networks.

#### Training methodology

The three areas of concentration for training methods seem to be the plan's initial broad distribution, methodical training, and technology training. It will be important to let all experts know what will be created once the firm's action plan has been defined. To establish a strong dedication to the initiative, senior firm executives should set the tone for “conviction” and “empowerment.” The workgroup must also demonstrate these high devotion rates, as they will be required to put in the most effort concerning time commitment and training. There must be explanation sessions and dialogues about the strategy to offer the whole team writing assistance for the measures to be taken. Priority must be given to BIM technique training. The technique must be taught conceptually, along with its scope and common difficulties. Then, explicit details should be provided regarding how the business will adjust in operational and strategic aspects, how every member would be allocated a job, and what effects the procedure will have. The inability to comprehend this well will significantly affect how well the implementation goes. The establishment of team training sessions and the distribution of background informational materials have been required. Technical pieces of training ought to be focused on the BIM role needs and chosen technology. To maximize employee curriculum, programme pieces of training should take into account prior professional experience. A tool may be created individually or in collaboration with a university to show that experts have completed scholarly certification if users do not already have a formal certificate from a university proving the knowledge they claim to possess (from, for example, expert fellowships or scholastic courses). To allocate the resources used for learning over time, learning should be carried out according to grade and predicated on execution progress. The business should deliver training services using its staff, third parties, or contracts made with public or private institutions. Pieces of training should ideally take place at the workplace. Content delivery ought to be intimately linked to the actual work done in the specified pilot project.

#### BIM working components regulation and development

Vignettes, parametric components (such as families), templates, information needs sheets, interruption recognition sheets, and other documents must be made and modified so that the agency has all the components of BIM systems at the pilot project started for the project's effective growth. The goal is to plot and display outputs in the BIM system (such as Revit) with identical features and properties as in 2D CAD (relating to the intended outcome in a plan). The workplace BIM Standard will outline the criteria for this.

## Evaluation and monitoring

### Knowledge monitoring

Professionals' knowledge acquisition should be closely scrutinized. To achieve this, examinations of knowledge on the application of programmes and methodologies that align with the progressive growth of information professionals learn will be administered. Such evaluations produced by the executing firm and by academic or technical institutions (house certification programmes) certify specialists and improve the operating team's effectiveness (for instance, in staff development for bidding). The organization's resources will determine the sort of accreditation.

### Parametric elements creation

A family architecture service will be offered to make modelling BIM systems easier. The business can streamline modelling processes and access project needs through this solution. The family architecture service has been intended to support the growth of BIM in the workplace and is not regarded as an element of the early installation costs.

### Assessment of plan and objectives compliance

All firm actions and processes must completely document decisions made within the system that the executing firm believes acceptable. The performance and indicator adherence should be noted in this document. Restructuring and reformulating planned actions that have not yet been fulfilled will enable the production of actions and plans.

### Resolving doubts

The organisation and the executing business must develop communication's active channels to determine how, when, and when to conduct evaluations of programme usage processes and technical features. To progressively make the organization self-sufficient, experts have been encouraged to educate independently and collaborate with teammates.

## Method validation

This paper presented a methodology for the implementation of BIM in the SEF. This technique contains procedures for assessment and prognosis, reconsideration of goals, needs identification, scheduling, and project tracking. It also clearly and precisely demonstrates how to conduct implementation. In addition, a brand-new hybrid AB-AVO framework has been created to assess the current approach. The exact needs for adopting BIM were outlined and accounted for the working team, technology and space allocation, BIM-focused workflows, protocols, and other crucial components for the execution's success. Further, training was among the most frequently mentioned critical enablers that might hasten an effective BIM adoption; the findings indicated that the methodology employed for BIM execution in this research was effectively trained. Moreover, in an attempt to explicitly define the firm's potential regarding BIM reorganization, the approach made obvious the tools to undertake the in-depth firm assessments and included stages for obtaining information on present objectives, procedures, and resources. On the other hand, to facilitate the integration of BIM in structural offices, professional unions should impose interoperability efficiency BIM as a basic reference in designing and implementing projects, making engineering companies qualify engineers. Moreover, it can encourage the resistance of these firms to adopt BIM for creating and managing data during the planning, designing, construction, and operations process, which in turn results in greater visibility, better decision-making, more sustainable options, and cost-savings on architecture, engineering, and construction projects.

### Reflection about the implementation of BIM and its limitation

In the early stages of BIM development, practitioners frequently overlook the alignment of their methodologies with established theoretical principles in decision-making and technology adoption. This oversight can result in a range of attitudes, both pessimistic and optimistic, introducing obstacles to the seamless integration of BIM in SEF. Conversely, making well-informed decisions regarding BIM adoption is likened to navigating a cause-and-effect relationship, emphasizing the crucial need for thorough reform at the state level within the construction industry, guided by effective leadership. This state-level reform is directly impacted by external factors, influencing not only the behaviors of practitioners but also shaping the formulation of BIM strategies at the state level. BIM, through performance monitoring, aims to assess and improve its functionality, streamlining the implementation process and significantly reducing variation orders. Consequently, the field study underscores the distinctive nature of these externalities, prompting skepticism regarding the suitability of universally accepted forms for evaluating industry readiness. Hence, a conceptual framework is introduced to enhance the reliability of BIM practices, with a specific focus on industry readiness during the pre-maturity phase. This framework is crafted for application in future studies, anticipating its role in guiding BIM developers during the pre-maturity stage to transform current outputs into dependable outcomes. Ultimately, this approach holds the potential to usher in a new era for engineering firms by influencing the adoption of a comprehensive work methodology spanning from planning and design to implementation. This, in turn, can enhance employee performance, contributing to an overall improvement in the quality and outlook of the final product.

## Funding

No funding was obtained for this study.

## CRediT authorship contribution statement

**Ibrahim F. Varouqa:** Conceptualization, Data curation, Formal analysis, Funding acquisition, Investigation, Methodology, Project administration, Software, Supervision, Visualization, Writing – original draft. **Moawiah A. Alnsour:** Conceptualization, Data curation, Formal analysis, Funding acquisition, Investigation, Methodology, Project administration, Resources, Validation, Visualization, Writing – review & editing.

## Data Availability

Data will be made available on request.
